# Establishment and Characterization of OS-MET-R-092: A Novel Patient-Derived Cell Culture from an Osteosarcoma Bone Metastasis

**DOI:** 10.3390/ijms262110540

**Published:** 2025-10-29

**Authors:** Veronica Giusti, Leonardo Fantoni, Monica Torsello, Giorgio Frega, Arianna Martinuzzi, Giulia Sbanchi, Caterina Dalrio, Enrico Lucarelli, Chiara Bellotti, Chiara Casotti, Elena Caddeo, Ania Naila Guerrieri, Simona Paglia, Claudia Maria Hattinger, Massimo Serra, Margherita Maioli, Marco Gambarotti, Stefania Benini, Luca Cattini, Davide Maria Donati, Toni Ibrahim, Laura Mercatali

**Affiliations:** 1 Osteoncology, Bone and Soft Tissue Sarcomas and Innovative Therapies Unit, IRCCS Istituto Ortopedico Rizzoli, 40136 Bologna, Italy; veronica.giusti@ior.it (V.G.); leonardo.fantoni@ior.it (L.F.); giorgio.frega@ior.it (G.F.); arianna.martinuzzi@ior.it (A.M.); giulia.sbanchi@irst.emr.it (G.S.); caterina.dalrio@gmail.com (C.D.); chiara.bellotti@ior.it (C.B.); chiara.casotti@ior.it (C.C.); elena.caddeo@ior.it (E.C.); anianaila.guerrieri@ior.it (A.N.G.); simona.paglia@ior.it (S.P.); claudia.hattinger@ior.it (C.M.H.); massimo.serra@ior.it (M.S.); toni.ibrahim@ior.it (T.I.); laura.mercatali@ior.it (L.M.); 2 Department of Pathology, IRCCS Istituto Ortopedico Rizzoli, 40136 Bologna, Italy; margherita.maioli@ior.it (M.M.); marco.gambarotti@ior.it (M.G.); stefania.benini@ior.it (S.B.); 3 Laboratory of Immunorheumatology and Tissue Regeneration, IRCCS Istituto Ortopedico Rizzoli, 40136 Bologna, Italy; luca.cattini@ior.it; 4 Orthopaedic Oncology Unit, IRCCS Istituto Ortopedico Rizzoli, 40136 Bologna, Italy; davidemaria.donati@ior.it; 5 Department of Biomedical and Neuromotor Sciences, University of Bologna, 40126 Bologna, Italy

**Keywords:** patient-derived cell culture, bone metastasis, osteosarcoma, ovoPDX, multi-drug resistance, preclinical model

## Abstract

Bone metastases from osteosarcoma occur in only 10% of patients, and related preclinical models are lacking. A patient diagnosed with pelvic osteosarcoma developed a metachronous scapular metastasis and was treated with multi-agent chemotherapy and surgery. Patient-derived tissue fragments (PDTFs) were obtained from leftover material after diagnosis and biobanking. PDTFs were grown on chick chorioallantoic membrane, establishing an in vivo-like predictive model. Additionally, we obtained a patient-derived cell culture, OS-MET-R-092, which has been maintained in vitro for nearly one year. OS-MET-R-092 cells were authenticated based on short tandem repeats and on their morphology when grown on commercial 3D scaffolds. Using U-2 OS and SaOS-2 as controls, we characterized growth, clonogenic potential, ability to form spheroids, migration, osteogenic differentiation, and expression of related genes. OS-MET-R-092 cells showed a low proliferation rate, impaired differentiation potential, and migratory abilities comparable to SaOS-2, while expressing higher levels of some MMPs and CD44. Functionally, OS-MET-R-092 cells demonstrated a resistant phenotype to doxorubicin, cisplatin, gemcitabine, and docetaxel, corroborated by higher expression of chemo-resistance-related genes. Collectively, OS-MET-R-092 represents a valuable tool for studying bone metastasis from osteosarcoma across various experimental settings and serves as the foundational building block for composite and translatable 3D models.

## 1. Introduction

Osteosarcoma (OS) is the most prevalent primary bone sarcoma, exhibiting a bimodal age distribution: a primary peak in adolescents and young adults (ages 10–24) and a secondary peak in older adults (over 60) [1]. Approximately 20% of patients present with detectable synchronous lung metastasis at diagnosis, which decreases overall survival from 70% to 20% [2]. Other than in the lung, OS has been anecdotally reported to metastasize to the kidney, skin [3], distant lymphnodes, and brain [4]. Bone secondary lesions are distinguished between skip metastasis [5], defined as secondary, synchronous lesions in the same bone, and metachronous bone metastases.

Despite their rarity, metachronous bone metastases represent an impactful event, leading to considerable morbidity, including pain, fractures, and impaired quality of life. These secondary lesions were first reported in 1966 by Lockshin and colleagues, who described a small case series of five patients diagnosed at their Institution within one year [6]. A decade later, Jeffree and colleagues went into pattern of OS metastasis, investigating a case series of 152 OS patients *plus* 43 autopsies [7,8]. They detected pulmonary metastases in 90% of patients, and extra-pulmonary metastases in 33% of patients. Extra-pulmonary metastases arose mainly in other bones at a median time of 9–10 months after first diagnosis of OS and were positively associated with local recurrences. The clinical–pathological relevance of metachronous bone metastases was explored in a longitudinal case series of OS patients at our Institution, IRCCS Istituto Ortopedico Rizzoli [9,10]. Among the 1148 OS patients treated between 1972 and 1999, 52 (4.5%) developed a bone metastasis as their first relapse, while 371 (32.3%) experienced metastases to the lung. Bone metastases appeared after a median time of 26 months and were associated with a higher incidence of local recurrences, confirming previous observations, and a poorer prognosis, in terms of 5-year post-relapse event-free and overall survival [9,10].

Currently, there is no consensus on the optimal management of metastatic OS patients. Most patients deemed amenable to complete surgical resection receive the same chemotherapeutic regimen as those with localized high-grade OS, which includes high-dose methotrexate (MTX), doxorubicin (DX), and cisplatin (CDDP) (i.e., MAP regimen). Further lines of treatment include high-dose ifosfamide (IFO) with or without etoposide, gemcitabine (GEM), and docetaxel (DOC) [11]. However, this regimen—and consequently, overall survival rates—has remained largely unchanged over the past three decades.

Up to date, over 100 distinct OS cell lines have been established and reported in the scientific literature globally [12,13]. Among them, models showing a lung-metastatic phenotype in vivo permitted researchers to unravel the molecular pathways and genetic determinants involved in lung metastasis [14,15], including matrix metalloproteinases (MMPs) [16] and CD44 [17,18]. On the contrary, as with many other rare conditions, no preclinical model is available for OS bone metastases, which contributes to the extremely limited understanding of the underlying molecular mechanisms [12,19]. The few existing papers highlighted the role of (i) bone morphogenetic proteins (BMPs), which can induce and activate various pathways in mesenchymal cells affecting bone formation, (ii) CD31, which is associated with migration between endothelial cells, and (iii) hepatocyte growth factor (HGF) reduced expression, failing to promote differentiation of OS cells [20].

In the present paper, we report the successful attainment of a patient-derived cell culture (PDCC) from an OS bone metastasis and its functional characterization in comparison to two established OS cell lines, chosen for their representativeness of different features [12,19]. SaOS-2 cells display slow growth and poor migratory abilities, are p53 null, and are capable of matrix mineralization. On the other hand, U-2 OS cells exhibit a more rapid growth and higher migratory abilities, harbor wild type p53, and have an expression profile more similar to osteoblasts, even if they do not mineralize the extracellular matrix (ECM) [19,21,22]. Though finite, OS-MET-R-092 cells may represent a useful tool for deepening our understanding of the biology of metachronous bone metastasis, while serving as the foundational building block for more composite and translatable 3D models.

## 2. Results

### 2.1. Clinical History

**Figure 1:**
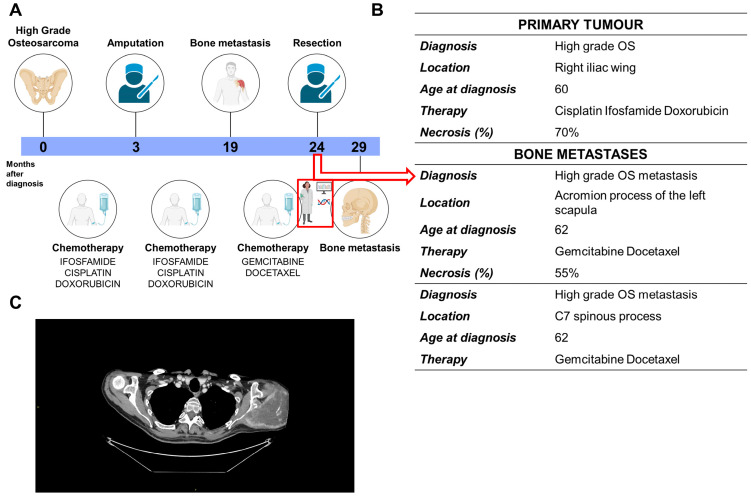
Patient’s clinical history. (**A**): Timeline of the clinical history of the patient; (**B**): summary of the clinical data of the patient. In A and B, the red arrow indicates the tissue specimen collected for translational research. (**C**): CECT, portal phase, showing the metastatic bone lesion of the acromion process—left scapula, represented by a swallowing and bone-remodeling lytic lesion in the left acromion process.

A 60-year-old man was diagnosed with a pelvic OS in 2022 (Figure 1A). He underwent neoadjuvant chemotherapy with DX, CDDP, and IFO (MTX was omitted because of age) (Figure 1B). The histological analysis of the primary tumor highlighted poor response (70% necrosis) and tumoral emboli inside blood vessels. The patient completed adjuvant chemotherapy 11 months after the initial diagnosis, using the same drugs. Eight months later, the patient reported pain in the left shoulder (Figure 1A,B). Chest and abdomen contrast-enhanced computed tomography (CECT) scans were negative for lung metastasis but showed a metachronous lytic bone metastasis (Figure 1C). A second-line chemotherapy was then started including GEM and DOC. After two cycles, the lesion was stable and the patient underwent surgery (24 months after first diagnosis). The histological analysis revealed a higher grade with respect to the primary OS and an even poorer response to chemotherapy (55% of necrosis) (Figure 1B). Fresh patient-derived tumor tissue was processed at our laboratory and anonymized under the acronym OS-MET-092. Twenty-nine months after OS diagnosis, the chest and abdominal CECT scans were still negative, but a magnetic resonance revealed a fracture in the spinous corpus of the 7th cervical vertebra (Figure 1A,B). The lesion was a plausible implication of the spread of disease and was accompanied by osteolysis in other vertebrae, as well. At the time of writing this paper, 16 months after the resection of scapular bone metastasis and 40 months after the diagnosis of OS, the patient was in follow-up with a cervical lesion and a suspicion of local recurrence; he was receiving treatment with zoledronic acid.

### 2.2. Establishment of OS-MET-092 Models

**Figure 2:**
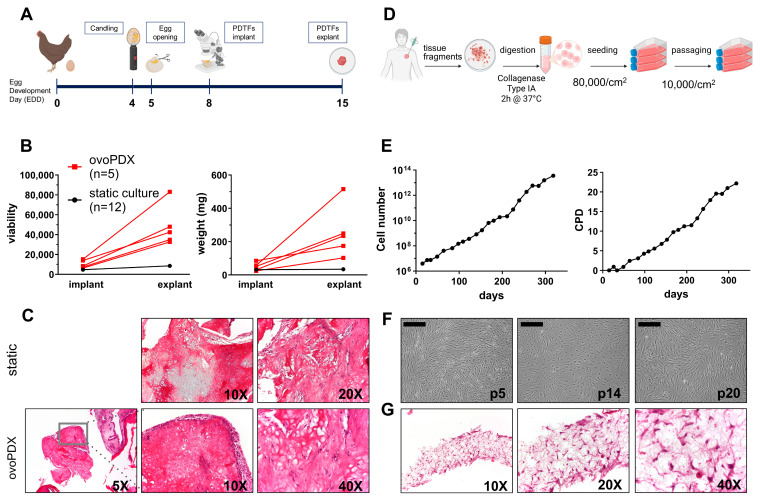
Establishment of OS-MET-092 models. (**A**): Schematical representation of the protocol for the establishment of ovoPDX; (**B**): comparison of viability (**left**) measured by PrestoBlue and weight (**right**) of OS-MET-092 PDTFs (i) cultured as ovoPDX and measured at implant (EDD8) and explant (EDD15). Each red point indicates a single fragment implanted; (ii) cultured under static conditions evaluated at the same time-points. The black points indicate the mean ± SEM of 12 fragments. (**C**): H/E staining of OS-MET-092 PDTFs cultured under static conditions in vitro or in ovo, as indicated. In each image, complete with objective magnification, it is possible to recognize atypic spindle epithelioid cells producing osteoid in a lace-like pattern; (**D**): schematic representation of OS-MET-092 tissue digestion and culture of OS-MET-R-092 cells; (**E**): growth curve of OS-MET-R-092 cells over time expressed as cell number (left) and CPD (right); (**F**): micrographs of OS-MET-R-092 cell morphology at passage p5, p14, and p20. All images 40X magnification (scale bar = 500 µm); (**G**): H/E staining of OS-MET-R-092 cells grown on a highly porous polystyrene scaffold showing atypical nuclei and prominent nucleoli, consistent with OS morphology. Objective magnification is indicated under each image.

The establishment and availability of appropriate disease models are pivotal in fostering research, especially within the context of rare and ultra-rare tumors. OvoPDXs (in ovo patient-derived xenografts) represent a simple in vivo-like system, which allows us to keep tissue explants alive outside the host environment. Moreover, the use of chick chorioallantoic membrane (CAM) assay has been proposed as a prognostic and predictive xenograft model for sarcomas, as the viability of the graft is inversely related to patient’s survival [23]. The rose-whitish semi-hard patient-derived tumor fragments (PDTFs) of the scapular metastasis were implanted on the CAM at egg development day 8 (EDD 8) (Figure 2A). In parallel, control samples *(n* = 12) were maintained in culture medium in 24-well plates without further handling (static conditions). During the experiment, one chick embryo died, and another one was contaminated. The remaining five PDTFs were engrafted on the CAM. At the endpoint (EDD 15), the PDTFs were embedded by the CAM and surrounded by the host vessels (Supplementary Figure S1). PDTFs grown on the CAM were harvested and measured; OS-MET-092 PDTFs had a bony consistency and showed increased weight and viability in respect to those cultured under static conditions (Figure 2B). Moreover, all the tissue specimens, either maintained in vitro or in ovo, retained a tissue morphology consistent with OS, characterized by atypical spindle epithelioid cells producing osteoid in a lace-like pattern (Figure 2C) [24,25].

To obtain a PDCC, 1 g of PDTFs was mechanically and enzymatically digested, yielding over 7.5 × 10^6^ cells, confirming the high viability of the bone metastasis (Figure 2D). The PDCC was named OS-MET-R-092 and was cultured for nearly a year. OS-MET-R-092 cells were routinary passaged once every two weeks, for more than 20 cumulative population doublings (CPD) and as many passages (Figure 2E). OS-MET-R-092 cells showed a spindle-like morphology and grew in a homogeneous monolayer, forming convoluted patterns at confluency. The PDCC retained the original morphology up to the 20th passage in vitro, with minor changes in size and increased vesiculation (Figure 2F). OS-MET-R-092 cells were significantly bigger and showed a wider size distribution compared to U-2 OS and SaOS-2 (average size: OS-MET-R-092: 33.33 μm; SaOS-2: 20.82 μm; U-2 OS: 17.12 μm; Supplementary Figure S2). This difference reflected the greater heterogeneity of PDCCs and could be associated with mechanisms of adaptation to grow in vitro or to other biological intrinsic characteristics (i.e., ploidy) [19]. OS-MET-R-092 cells were authenticated in comparison to the originating tumor tissue by short tandem repeats (STR) profiling (Table 1). The analysis highlighted marginal differences in three loci (TH01, vWA, and D18S51), which may be due to a fraction of normal cells in the patient’s tissue, no longer detectable in the PDCC. Moreover, an expert pathologist recognized OS-MET-R-092 cells as malignant OS due to their atypical nuclei and prominent nucleoli. The morphologic evaluation was conducted on OS-MET-R-092 cells grown on a highly porous polystyrene scaffold. Other than their malignant nature, the hematoxylin/eosin (H/E) staining revealed the complete colonization of the 3D scaffold with the cells spreading from the top to the bottom and from the center to the edges, reflecting their high propensity to migrate (Figure 2G).

### 2.3. Characterization of OS-MET-R-092 Cells

**Figure 3:**
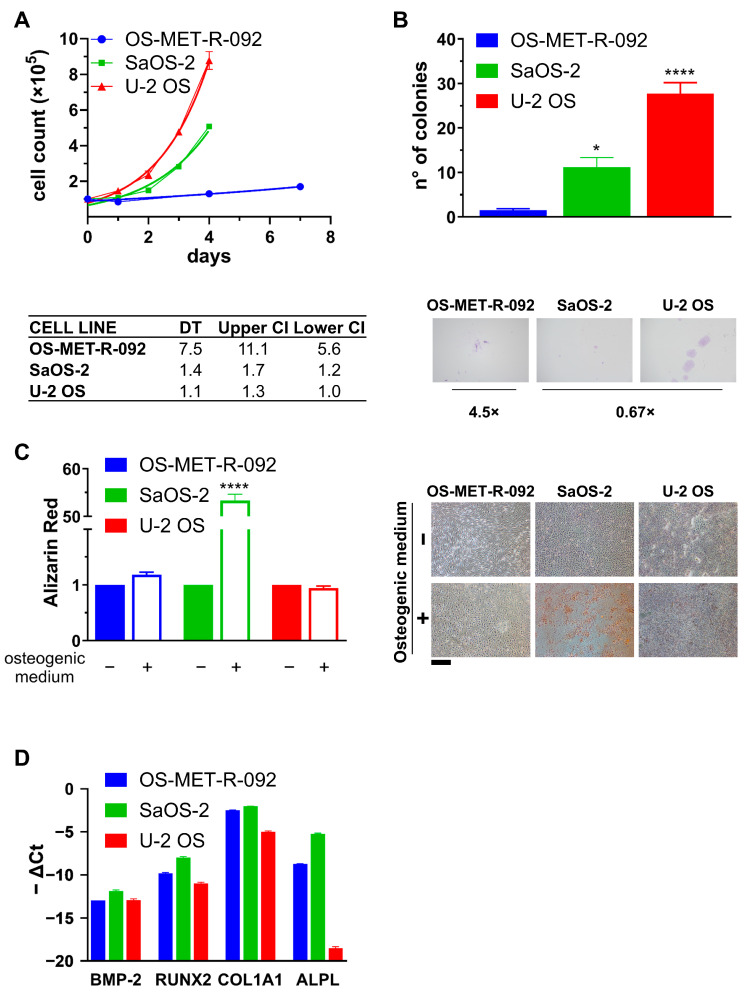
Characterization of OS-MET-R-092 cells. The phenotype of OS-MET-R-092 cells (blue) was compared to two reference established OS cell lines, SaOS-2 (green) and U-2 OS (red). Dots and bars represent the mean ± SEM. Statistical analysis: all comparisons versus OS-MET-R-092 * *p* < 0.05; **** *p* < 0.0001. (**A**): Proliferation assay: top: each symbol represents the cell count at the indicated time points, continuous lines indicate the interpolated exponential growth curves. Bottom: doubling time (DT) values and the relative 95% Confidence Intervals (CI) obtained from the interpolation of the exponential growth curve. (**B**): Clonal efficiency: top: each bar represents the number of colonies formed upon seeding of 100 cells. Bottom: micrographs of representative fields (4.5× or 0.67×). (**C**): Osteogenic differentiation: **left**: Each bar represents the relative quantification of total calcium deposited in control (−; solid) or osteogenic medium (+; empty). **Right**: representative fields of Alizarin Red staining. Scale bar = 500 µm. (**D**): Expression of osteogenesis-related genes by qRT-PCR. Bars represent the opposite of the ΔCt (Ct_gene_ − Ct_ACT_), and β-actin was employed for normalization purposes.

OS-MET-R-092 cells were characterized in comparison to U-2 OS and SaOS-2 cells, two commercially available OS cell lines, the former recognized as aggressive and metastatic, the latter as indolent and osteo-competent [19,21]. Firstly, cells were compared for their proliferation in 2D culture conditions. While the two commercially available cell lines doubled in roughly 24 h (1.4 days for SaOS-2 and 1.1 for U-2 OS), OS-MET-R-092 cells were significantly slower and the population doubled in 7.5 days (Figure 3A). U-2 OS showed a greater clonogenic ability compared to SaOS-2, whereas OS-MET-R-092 showed no clonogenic potential (Figure 3B). When higher numbers of cells were seeded, OS-MET-R-092 tended to grow as a loose monolayer, rather than forming colonies (Supplementary Figure S3A). The slower growth, common to other PDCCs, reflects the lower adaptation to in vitro growth and therefore the greater faithfulness of these models [19,26].

The three cell lines were also compared in terms of osteogenic potential. As expected, SaOS-2 cells exhibited extensive deposition of mineralized matrix, as evidenced by intense Alizarin Red staining (Figure 3C) [19,21]. In contrast, U-2 OS cells showed minimal staining, consistent with their limited capacity for ECM mineralization (Figure 3C). OS-MET-R-092 cells underwent a notable morphological change, becoming wider and stocky with a diaphanous cytoplasm, consistent with osteogenic induction (Supplementary Figure S3B). Nevertheless, Alizarin Red staining revealed no detectable calcium deposits, as observed in U-2 OS cells (Figure 3C). Alongside the functional characterization, we analyzed the expression of osteogenesis-related genes, included in the canonical BMP-2 signaling pathway. BMP-2 itself was minimally expressed in all OS cell lines, in accordance with the literature and its inhibitory role in OS (Figure 3D) [27,28]. In line with the functional observation and previously reported data [27], COL1A1, RUNX2, and ALPL were most highly expressed by SaOS-2 cells and minimally by U-2 OS cells; OS-MET-R-092 cells exhibited an intermediate expression, more closely resembling the SaOS-2 profile (Figure 3D). This expression pattern was also consistent with the histopathological analysis of the tissue specimen, characterized by sparse and focal Alizarin Red staining (Supplementary Figure S3C). Altogether, the molecular analysis corroborated the functional observations, indicating that OS-MET-R-092 cells can undergo osteogenic induction but are unable to mineralize the ECM.

**Figure 4:**
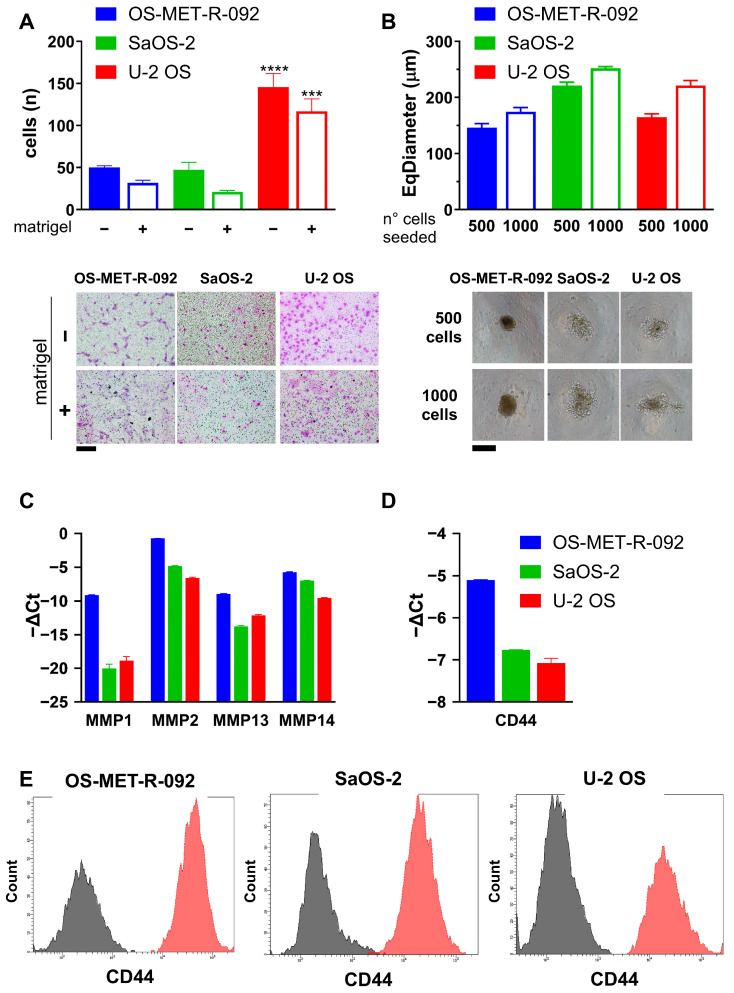
Invasive phenotype of OS-MET-R-092 cells. The phenotype of OS-MET-R-092 cells (blue) was compared to two reference established OS cell lines, SaOS-2 (green) and U-2 OS (red). Dots and bars represent the mean ± SEM. Statistical analysis: all comparisons versus OS-MET-R-092; *** *p* < 0.001; **** *p* < 0.0001. (**A**): Migration and invasion assay: **top**: each bar represents the number of cells crossing the transwell membrane in absence (solid) or presence (empty) of coating with Matrigel. **Bottom**: Micrographs of representative fields. Scale bar = 200 µm. (**B**): Spheroid formation: top: each bar represents the diameter of spheroids formed upon seeding of 500 (solid) or 1000 (empty) cells in 96-well ultra-low attachment (ULA) plates for 4 days. Bottom: micrographs of representative spheroids. Scale bar = 200 µm. (**C**,**D**): Expression of matrix-related genes by qRT-PCR. Bars represent the opposite of the ΔCt (Ct_gene_ − Ct_ACT_), and β-actin was employed for normalization purposes. (**E**): Cytofluorimetric analysis of CD44 expression. The overlays show the profile of unstained cells in grey and stained ones in red.

The migratory and invasive abilities of OS-MET-R-092 cells were also investigated (Figure 4A and Supplementary Figure S4A). All the three cell lines were able to cross the transwell membrane and, as expected, they exhibited a reduced migration in the presence of Matrigel. OS-MET-R-092 cells displayed a migratory and invasive behavior similar to that of SaOS-2, and inferior to U-2 OS cells, which are known for their higher motility and invasiveness (Figure 4A) [29]. Analogously, in wound healing assays, U-2 OS cells required less than 24 h to close the scratched area of a confluent monolayer, and exhibited markedly higher motility than SaOS-2 and OS-MET-R-092 cells, which needed more than 40 h (Supplementary Figure S4A). Of note, the ratio between the number of migrating (without Matrigel; −) and invading (with Matrigel; +) cells was significantly lower for OS-MET-R-092 cells compared to SaOS-2. We also evaluated the ability of OS-MET-R-092 cells to grow under 3D non-adherent conditions. Both OS-MET-R-092 and the reference cell lines demonstrated the ability to form spheroids (Figure 4B). Notably, OS-MET-R-092 generated compact and morphologically homogeneous spheroids (shape factor: 0.920; roughness: 1.093; Supplementary Figure S4B), which exhibited a decrease in diameter over time (Supplementary Figure S4B) and rapidly developed a darker core. In contrast, spheroids obtained from SaOS-2 and U-2 OS appeared bigger in size, but displayed irregular and less cohesive structures, characterized by progressive disaggregation and peripheral fragmentation. The observed morphological difference was quantitatively corroborated by lower shape factor values and higher surface roughness, which may reflect variations in the composition of the ECM (Supplementary Figure S4B). Both the invasive potential and the morphology of the spheroids can be attributed to a matrix-remodeling phenotype, which OS-MET-R-092 cells likely acquired due to their origin from metastatic tissue. Hence, we evaluated the expression of migration-related genes such as MMPs [16], as well as CD44, a cell surface receptor which binds several ECM proteins and is involved in the metastatic process [17,18]. MMP1, MMP2, MMP13, and MMP14 were all up-regulated in OS-MET-R-092 cells in respect to both U-2 OS and SaOS-2 cells (Figure 4C). Analogously,, CD44 was up-regulated in OS-MET-R-092 cells, as confirmed by both gene expression analysis and flow cytometry (Figure 4D,E), highlighting its potential role in the migratory and invasive behavior of these cells.

### 2.4. OS-MET-R-092 Cells Display a Multi-Drug Resistant Phenotype

**Figure 5:**
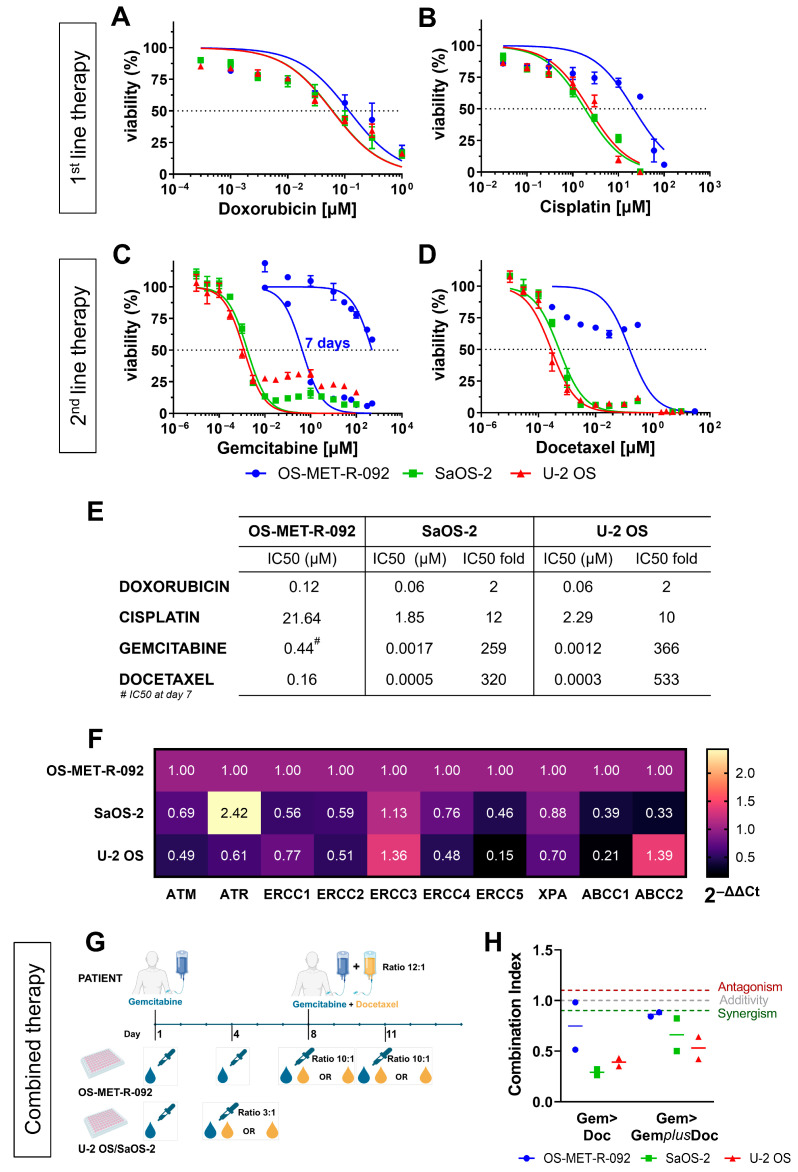
OS-MET-R-092 cells display a chemo-resistant phenotype. Non-linear transformation of dose–response curves of OS-MET-R-092 (blue), U-2 OS (red), and SaOS-2 (green) in response to DX (**A**), CDDP (**B**), GEM (**C**), and DOC (**D**) fitted using GraphPad Prism. Each curve represents the mean of at least three independent biological replicates. The table (**E**) reports the IC50 values for the three cell lines and the fold change in IC50 between OS-MET-R-092 and SaOS-2 or U-2 OS. (**F**) Expression of genes involved in chemo-resistance. The heatmap plot shows the comparison of gene expression between OS-MET-R-092 and the SaOS-2 and U-2 OS cell lines for the selected genes. The plot was generated using the 2^−ΔΔCt^ values, with OS-MET-R-092 serving as the reference sample. β-actin was employed for normalization purposes. (**G**) Schematic representation of combined treatment in the patient and in vitro. (**H**) Combination index (CI) for the associations of GEM>DOC and GEM>GEM*plus*DOC for OS-MET-R-092 (blue), U-2 OS (red), and SaOS-2 (green). CI calculated according to the equation of Chou–Talalay: synergistic CI ≤ 0.90, antagonistic CI ≥ 1.10 and additive 0.90 < CI < 1.10. Each symbol represents a biological replicate and the line represents the mean.

Modelling rarer entities of sarcomas is essential for designing and validating novel drugs. Since the PDCC originated from a metastatic patient previously treated with multi-agent chemotherapy, it was conceivable that OS-MET-R-092 cells may be resistant to one or more first-line drugs. Therefore, we evaluated their sensitivity towards the first- and the second-line drugs used for the patient’s treatment (Figure 1A). OS-MET-R-092 cells were slightly more resistant than U-2 OS and SaOS-2 to DX (Figure 5A,E). Notably, a tenfold increase was observed in the half-maximal inhibitory concentration (IC50) for CDDP between OS-MET-R-092 and the two reference cell lines (Figure 5B,E). The first-line therapy also included IFO, which was not tested in vitro, as it is a prodrug requiring enzymatic activation in vivo. The second-line included GEM and DOC, which were first evaluated as single agents in vitro. Over 96 h, OS-MET-R-092 cells were unaffected by the treatment with GEM, with around 50% viability at the limit dose of 500 μM (Figure 5C). Considering the cytostatic nature of the drug, we extended the treatment time. At 7 days, it was possible to calculate the IC50 for GEM in OS-MET-R-092 cells, but the value was still two orders of magnitude higher in respect to the reference cell lines (Figure 5C,E). Regarding DOC, the viability of OS-MET-R-092 cells remained above 50% up to 1 μM and dropped sharply at 10 μM (Figure 5D). For this reason, accurate fitting of the IC50 curve was not possible and the calculated IC50 value should be considered only as an estimate (Figure 5E). Overall, OS-MET-R-092 appeared more resistant than U-2 OS and SaOS-2 to every single chemotherapeutic drug employed in the first- and second-line of therapy.

As an initial investigation into the molecular basis underlying the chemo-resistant phenotype of OS-MET-R-092 cells, we assessed the expression of ABCB1 protein, a well-established marker of multi-drug resistance [30]. OS-MET-R-092 cells expressed low levels of ABCB1, comparable to those observed in SaOS-2 and U-2 OS, indicating that this membrane transporter did not substantially contribute to the drug-resistant phenotype of these cells (Supplementary Figure S5). To gain deeper insight, we analyzed the expression of a broader panel of genes involved in the nucleotide excision repair pathway (NER), in the recognition of damage to DNA (ATM and ATR), as well as other ABC transporters associated with multi-drug resistance. Overall, OS-MET-R-092 cells exhibited a higher expression of most of the investigated genes compared to the chemo-sensitive cells (Figure 5F). Although individual changes in gene expression were modest, their combined up-regulation suggested a trend towards a more efficient damage repair capacity. In addition, CD44, which was up-regulated in OS-MET-R-092 (Figure 4D,E), was alsoreported to play a role in chemo-resistance [17,31,32], further supporting this phenotype.

Since the patient’s second line of therapy consisted of cycles of GEM administered at day 1, followed by the co-administration of GEM and DOC at day 8 in a 12:1 ratio, we sought to replicate this schedule in vitro (Figure 5G,H). The administration of GEM, followed by either DOC alone (GEM>DOC) or GEM combined with DOC (GEM>GEM*plus*DOC) resulted in a synergistic effect on day 11, as confirmed by a combination index (CI) < 0.9 (Figure 5G). The same approach was used on the reference cell lines, adjusting the experimental schedule according to their higher growth rate and the drug ratio according to their IC50 (Figure 5H). Consistently with the results obtained for the OS-MET-R-092, the GEM-DOC sequential administration exhibited a synergistic effect in both the reference cell lines (Figure 5G).

OS-MET-R-092 cells showed a low proliferation rate, impaired differentiation potential, and migratory abilities comparable to SaOS-2, while expressing higher levels of some MMPs and CD44. Functionally, OS-MET-R-092 cells demonstrated a resistant phenotype to doxorubicin, cisplatin, gemcitabine, and docetaxel, corroborated by higher expression of chemo-resistance-related genes. This body of evidence indicates that OS-MET-R-092 may represent a reliable preclinical model that could help elucidate the molecular mechanisms underlying the development of osteosarcoma bone metastases and guide the identification of innovative therapeutic strategies for metastatic OS patients.

## 3. Discussion

Metastases represent the primary cause of death for patients with OS [15]. Nearly half of OS patients develop lung metastasis, decreasing survival to 30–40% [2]. Bone metastases, however, are an uncommon event in OS, occurring in less than 10% of patients, but their appraisal is equally associated with poorer prognosis [9,10]. Surgical resection of metastases can improve survival, but systemic treatments have shown limited efficacy in the relapsed setting [33]. Notably, there is no consensus on the chemotherapeutic regimen to follow for bone metastatic patients. Despite major advances in biomedical research, the successful translation of preclinical findings into effective clinical therapies remains limited, partly due to the scarcity of faithful preclinical models that accurately capture the biological complexity of OS, particularly in advanced and therapy-resistant stages [12,19].

In this study, we exploited PDTFs from an OS bone metastasis as a preclinical model with prognostic and predictive value. We evaluated their ability to grow under static conditions in plastic well plates, as well as on the CAM of chick embryos, which provides an in vivo-like environment. PDTFs seeded on the CAM demonstrated sustained growth over time and exhibited higher viability compared to those maintained under static conditions, while preserving the original tissue morphology and structural integrity (Figure 2A–C). OvoPDXs have successfully been established in a variety of sarcomas offering insights into faithfulness, viability, Ki67 proliferation index, necrosis, invasive potential, fibroblastic and vascular ingrowth by the host [23,34,35]. Research in carcinoma has also demonstrated the applicability of ovoPDX for studying anti-tumor, anti-angiogenic, and anti-metastatic molecules [36]. OvoPDX could, hence, be exploited as a low-cost, ethical, time-effective and faithful in vivo-like model for the validation of targeted molecules identified upon molecular characterization [14]. Moreover, the successful engraftment of OS-MET-092 PDTFs on the CAM could reflect its aggressive behavior in the patient. Indeed, shortly after the resection of the scapular bone metastasis, the patient experienced another recurrence in the C7 spinous process (Figure 1). The direct use of PDTFs allows preservation of all the architectural and cellular components of the original tissue, although reproducibility is limited by intra-tumor heterogeneity and by the availability of initial material [14]. On the other hand, the establishment of PDCCs from surgical specimens enables the development of more suitable models for drug screenings and molecular analysis [24,25]. For these reasons, we sought to establish a PDCC, designated OS-MET-R-092, from the same surgical specimen. The high cellular yield upon enzymatic digestion is consistent with the low level of necrosis (55%) (Figure 1B). The cells retained their original spindle-like morphology, and continuous culture was maintained for nearly a year, encompassing more than 20 in vitro passages and a comparable number of CPD (Figure 2E). Accordingly, OS-MET-R-092 displayed a very long DT and an extremely low clonogenic ability (Figure 3A,B). The slow growth rate and limited lifespan are common features of patient-derived OS cell lines, making them challenging to work with due to their low throughput, yet valuable because of their biological relevance [19,37,38]. OS has the highest number of available patient-derived cellular models among sarcomas, a characteristic that contrasts to the overall scarcity of models for most other sarcoma subtypes [12,19]. Despite that, to the best of our knowledge, OS-MET-R-092 represents the first reported PDCC established from an OS metastatic lesion in bone tissue. This uniqueness, together with the known advantages of using cells at low passages, compensates for the failed immortalization of the culture [38,39]. The high fidelity of OS-MET-R-092 PDCC was confirmed by both STR analysis and morphological identification of 3D-cultured cells as osteosarcoma (Table 1 and Figure 2G). Moreover, the ability of OS-MET-R-092 cells to grow and populate a porous polystyrene scaffold offered the opportunity to generate advanced models mimicking the spatial complexity of the original tissue [13,40]. OS-MET-R-092 was subsequently characterized in comparison to U-2 OS and SaOS-2 (Figure 3 and Figure 4), two well-established immortalized OS cell lines known to be the former aggressive and metastatic, the latter indolent and osteo-competent [19,21]. When the osteogenic differentiation potential was evaluated, OS-MET-R-092 cells failed to mineralize ECM, despite showing morphological changes (Figure 3C and Supplementary Figure S3B). The analysis of genes related to the BMP-2 pathway revealed that OS-MET-R-092 cells exhibited an expression profile consistent with the functional observations, showing intermediate levels of RUNX2, COL1A1, and ALPL compared to the reference cell lines (Figure 3D), corroborating the limited osteo-competence of the PDCC. In OS, impaired osteogenic differentiation is associated with increased proliferation and tumor aggressiveness; this behavior could also be ascribed to OS-MET-R-092 metastatic derivation [27].

The origin of OS-MET-R-092 from a metastatic tissue may suggest that it already acquired matrix-remodeling and invasive abilities. OS-MET-R-092 behaved similarly to SaOS-2 in migration and invasion assays, showing a lower migration ability compared to U-2 OS (Figure 4A; Supplementary Figure S4A). However, OS-MET-R-092 migratory abilities were less impacted by the addition of Matrigel in respect to SaOS-2 cells and this could reflect the activation of matrix-remodeling mechanisms. Moreover, OS-MET-R-092 successfully formed compact and morphologically uniform spheroids (Figure 4B), features likely associated with enhanced secretion of ECM proteins and the establishment of tight intercellular junctions [41]. On these bases, we explored the expression of invasion-related genes. We found several MMPs up-regulated in OS-MET-R-092 cells in respect to both SaOS-2 and U-2 OS (Figure 4C). MMPs are a family of proteolytic enzymes implicated in matrix-remodeling and cancer progression. The association of MMP1, MMP2, MMP13, and MMP14 with migration and metastasis is well established in the literature both at experimental [42,43,44] and clinical levels [16,45,46,47,48,49]. CD44 is a transmembrane protein, also interacting with MMPs, with a well-established role in tumor progression, invasion, and metastasis in OS [50,51]. In the literature, CD44 is recognized as a useful diagnostic and prognostic marker in OS [52,53,54]; moreover, its co-expression with MMP2 has been correlated with worse prognosis and lung metastasis [45]. The concomitant up-regulation of MMPs and CD44 in OS-MET-R-092 likely underlies its aggressiveness.

PDCCs represent the cornerstone of translational research and precision oncology [55]. Patient history suggested that OS-MET-R-092 cells could be resistant to one or more chemotherapeutic drugs. Hence, our PDCC was tested for its sensitivity to the first- and second-line chemotherapeutics administered to the patient: DX and CDDP during the neoadjuvant and adjuvant phase, and GEM and DOC after relapse to bone (Figure 1A). OS-MET-R-092 cells were moderately resistant to DX, while treatment with CDDP resulted in an IC50 approximately tenfold higher than that of the other OS cell lines (Figure 5A,B,E). This level of resistance is comparable to U-2 OS/CDDP 1 μg and SaOS-2/CDDP 1 μg resistant cell lines reported by Pasello et al. [56]. Moving to second-line treatments, OS-MET-R-092 cells showed marked resistance to both GEM and DOC (Figure 5C–E). Notably, when tested in combination to mimic the standard therapy administered to the patient, the treatment acted synergistically (Figure 5G,H). Nevertheless, the limited efficacy of second-line drugs may be related to the further metastasis diagnosed in the patient (Figure 1B). Overall, OS-MET-R-092 appeared more resistant than U-2 OS and SaOS-2 to every single chemotherapeutic drug employed in the first and secondline of therapy. Upon analysis of ABC transporters involved in multi-drug resistance, OS-MET-R-092 did not exhibit a higher expression of ABCB1, which is implicated in resistance to all the four drugs analyzed [30]; however, OS-MET-R-092 cells over-expressed ABCC1 compared to U-2OS and SaOS-2. The low expression of ABCB1 is consistent with the moderate resistance to DX, whereas higher expression of ABCC1 was observed in cell lines resistant to the main drugs used in OS treatment, compared to sensitive cell lines [57]. Broadening our analysis to other resistance-related genes, OS-MET-R-092 cells exhibited up-regulation of ERCC1, ERCC2, ERCC4, and ERCC5 compared to drug-sensitive cells (Figure 5F). Indeed, CDDP-resistant cell lines have been reported to exhibit higher expression of the ERCC gene family [58]. In addition, CD44, up-regulated in OS-MET-R-092 cells, is also involved in chemo-resistance [17,31,32]. Altogether, OS-MET-R-092 exhibits a tendency toward enhanced DNA repair activity in response to chemotherapy-induced damage, accompanied by increased drug efflux capacity. This preliminary molecular characterization further supports the relevance of this model, as it may contribute to elucidating the mechanisms underlying multi-drug resistance and to guiding the identification of innovative therapeutic strategies for patients with metastatic osteosarcoma.

In the current study, we successfully established and characterized a novel and unique PDCC from a bone metastasis of OS. This primary cell line showed very low proliferation yet moderate invasive abilities and displayed intrinsic resistance to most first- and second-line drugs in use for OS. The phenotypic observations were corroborated by the up-regulation of key genes involved in osteogenesis, invasion, and drug-resistance. Beyond standard 2D culture conditions, we were able to generate spheroids and to culture OS-MET-R-092 cells on commercial 3D scaffolds. Importantly, we succeeded in growing PDTFs as ovoPDXs, in the context of an in vivo-like system. Notwithstanding the limitations due to finite nature of OS-MET-R-092 and to the preliminary nature of the molecular profilingof the PDCC, ovoPDX and 3D scaffold-based models, OS-MET-R-092 and its characterization provide a foundation for studying bone metastasis from OS in diverse experimental settings. Moreover, we propose OS-MET-R-092 as a first building block for more composite and translatable 3D models ultimately aimed at uncovering the molecular mechanisms underlying OS metastasis, drug-resistance, and identifying innovative therapeutic strategies for metastatic patients.

## 4. Materials and Methods

### 4.1. Patient’s History

The patient, a 60-year-old man, was diagnosed with a high-grade pelvic OS, followed by a metachronous bone metastasis in 2024. The bone metastasis was surgically resected with curative intent (see Figure 1C). The tumor tissue exceeding diagnostic need was (i) lively stocked; (ii) snap-frozen for molecular analysis (i.e., authentication); (iii) implanted for the establishment of ovoPDX (see Section 4.11); and (iv) enzymatically digested (see Section 4.2) to establish the PDCC characterized in the present study. The tumor specimen was anonymized under the name OS-MET-092. The study protocol (EX-OVO) was approved by the local Ethics Committee (AVEC n. 552/2022/Sper/IOR); written informed consent was obtained from the patient before the enrollment.

### 4.2. Establishment of the Cell Line and Authentication

OS-MET-092 fragments were digested to obtain a PDCC, named OS-MET-R-092. The tissue was minced with sterile scissors and enzymatically digested in a solution of 2 mg/mL collagenase type IA (Sigma-Aldrich, St. Louis, MO, USA; C2674) at 37 °C with gentle shaking. After 2 h, the enzymatic reaction was blocked and the suspension was filtered through a 100 µm nylon mesh strainer. The cells were seeded at a density of 8 × 10^4^ cells/cm^2^ and subcultured every two weeks at a density of 1 × 10^4^ cells/cm^2^. All experiments involving OS-MET-R-092 were performed using low-passage cells, specifically between passages 5 and 15.

The obtained PDCC, OS-MET-R-092, was characterized in comparison with SaOS-2 (ATCC, HTB-85, RRID: CVCL_0548) and U-2 OS (ATCC, HTB-96, RRID: CVCL_0042). All cell lines were cultured in DMEM high glucose (HG) (Euroclone, Pero, Italy; ECB7501L) supplemented with 10% FBS heat-inactivated (HI) (Euroclone, ECS5000L), 1% GlutaMax (Thermo Fisher Scientific, Pailsley, UK; 35050038) and 1% Penicillin/Streptomicin (Euroclone, Pero, Italy; ECB3001), and maintained at 37 °C in a humidified atmosphere containing 5% CO_2_. Cells were authenticated by STR profiling using the Applied Biosystems AmpFLSTR Identifier Plus PCR Amplification Kit by Eurofins Genomics and routinely tested for mycoplasma contamination using the MycoAlert Mycoplasma Detection Kit (Lonza, Rockland, ME, USA; LOLT07418).

Cell number and viability were assessed using the Countess II FL Automated Cell Counter (Invitrogen, Bleiwijk, The Netherlands; AMQAF1000). The population doubling (PD) for each passage was calculated using the formula: *PD* = log_2_ (*N*_1_/*N*_0_) where *N*_0_ is the number of cells seeded and *N*_1_ is the number of cells harvested at confluency. CPD was calculated as the sum of PDs across passages. To calculate the DT, 1 × 10^5^ cells/well were seeded in 6-well plates. Cells were harvested and counted over a week. This assay was conducted in biological triplicate.

When required by the protocol, micrographs were taken using an inverted Nikon Eclipse TE2000-U microscope (Nikon Europe B.V., Amstelveen, The Netherlands; RRID:SCR_023161), equipped with a Nikon DS-Vi1-U3 CCD camera, and analyzed with NIS-Elements Basic Research software (Nikon Europe B.V., Amstelveen, The Netherlands; RRID:SCR_002776).

### 4.3. 3D Culture on Commercial Scaffolds

To validate OS-MET-R-092 as OS, cells were grown on Alvetex scaffolds (Reprocell, Glasgow, UK; AVP005-12) and their morphology was evaluated by an expert pathologist. Cell handling and seeding, as well as downstream processing for viability testing and histological analysis, were performed according to the manufacturer’s protocol.

### 4.4. Two-Dimensional Clonogenic Assay

Cells (100) were seeded in 6-well plates in technical triplicate. For OS-MET-R-092, we also seeded 500, 1250, and 5000 cells. Colony formation was assessed after 11 days for SaOS-2 and U-2 OS, and after 14 days for OS-MET-R-092. Cells were fixed in methanol for 10 min, followed by staining with Giemsa solution (Sigma-Aldrich, St. Louis, MO, USA; 48900) for 10 min at room temperature. Colonies were counted and compared across the different cell lines. This experiment was performed in biological duplicate.

### 4.5. Osteogenic Differentiation Assay

Cells (3 × 10^4^ cells/well) were seeded in 6-well plates in α-MEM medium (Euroclone, Pero, Italy; ECM0849L) supplemented with 2% FBS. A differentiation medium containing β-glycerophosphate (10 mM), dexamethasone (100 nM), and ascorbic acid (284 µM) (Sigma-Aldrich, St. Louis, MO, USA; G9891, D4902, A4544) was added to the experimental wells twice a week. After 14 days, cells were fixed in 70% ethanol for 1 h and stained with a 40 mM Alizarin Red S solution (pH 4.0–4.2) (Sigma-Aldrich, St. Louis, MO, USA; A5533) for 15 min at room temperature to detect mineralized matrix. Bound Alizarin Red was solubilized with 10% acetylpyridinium chloride (Sigma-Aldrich, St. Louis, MO, USA; C9002) in 10 mM sodium phosphate buffer (pH 7.0) (Sigma-Aldrich, St. Louis, MO, USA; S8282) and optical density was measured at 562 nm using a microplate reader (Synergy HT, BioTek, Winooski, VT, USA; RRID:SCR_020536).

### 4.6. Invasion and Migration Assays

The migratory and invasive capacities were assessed using 6.5 mm Transwell inserts with 8.0 µm pore polycarbonate membranes (Corning, Bedford, MA, USA; 3422), either uncoated (migration assay) or coated with Matrigel (invasion assay; Corning, Bedford, MA, USA; 354230). For the invasion assay, the upper side of each insert was coated with 100 µL of a 200 µg/mL Matrigel solution in DMEM HG and incubated at 37 °C for 1 h to allow solidification. For both assays, 1.5 × 10^4^ cells were seeded in the upper chamber of the transwell in 100 µL of DMEM HG supplemented with 0.1% *w*/*v* bovine serum albumin (BSA) (Sigma-Aldrich, St. Louis, MO, USA; A7888), while the lower chamber contained DMEM HG with 10% FBS HI as a chemoattractant. After 18 h of incubation, cells on the lower surface of the membrane were fixed in methanol and stained with Giemsa solution as described above (see Section 4.4). The number of migrated or invaded cells was counted in at least ten fields per transwell. The experiment was performed in technical triplicate and biological duplicate.

### 4.7. Spheroid Formation Assay

Cells (500 and 1000 cells/well) were seeded in ULA 96-well plates (Corning, Bedford, MA, USA; CLS4520). Spheroid EqDiameter [µm], shape factor, and roughness were measured at day 4 and day 7 using Nikon’s Spheroid Sizing and Morphology Analysis tool. The experiment was conducted in technical decuplicate and biological duplicate.

### 4.8. Gene Expression Analyses

Expression level of genes involved in osteogenesis, metastasis, and chemo-resistance was assessed by quantitative Reverse Transcriptase Polymerase Chain Reaction (qRT-PCR). Total cellular RNA was extracted using AllPrep DNA/RNA mini kit (Qiagen, Hilden, Germany; 80204) following the manufacturer’s instructions. The obtained RNA was quantified through Nanodrop ONE (Thermo Fisher, RRID:SCR_023005) and 500 ng were reverse transcribed using iScript cDNA synthesis kit (Bio-Rad Laboratories, Hercules, CA, USA; 170-8840). Gene expression analyses were performed through the real-time PCR CFX OPUS 96™ detection system (Bio-Rad Laboratories, Hercules, CA, USA) using 5 ng of cDNA. Both SYBR Green (SsoAdvanced Universal SYBR^®^ Green Supermix; Bio-Rad Laboratories, Hercules, CA, USA; 1725270) and TAQMAN approaches (SsoAdvanced Universal Probes Supermix; Bio-Rad Laboratories, Hercules, CA, USA; 1725280) were employed. Detailed information on primers and probes are described in Table 2. The data were analyzed following the 2^−ΔΔCt^ method [59]. β-actin was employed for normalization and OS-MET-R-092 as the reference sample.

### 4.9. Drug Treatment

Drugs used in the experiments were DX (Sigma-Aldrich, St. Louis, MO, USA; D1515), CDDP (Sigma-Aldrich, St. Louis, MO, USA; P4394), DOC (Hikma Pharmaceuticals, London, UK; 1065001), and GEM (Sandoz, Milano, Italy; 23779061). DX and CDDP were dissolved in sterile water and physiological solution at the concentration of 2 mg/mL and 500 µg/mL, respectively, and stored at −20 °C. Stock solutions of DOC (20 mg/mL) and GEM (40 mg/mL) were stored at +4 °C. Appropriate and fresh dilutions of the afore-mentioned drugs were made in culture medium prior to use.

Cells (1 × 10^4^ OS-MET-R-092/SaOS-2 cells, and 2 × 10^3^ U-2 OS) were seeded in 96-well plates and treated with either single agents or drug combinations 24 h after seeding. Cell viability was determined using PrestoBlue (Invitrogen, Bleiwijk, The Netherlands; A13262) after 96 h of treatment measuring fluorescence at 530/590 nm using a microplate reader (Synergy HT, BioTek Instruments Inc., Winooski, VT, USA; RRID:SCR_020536). The efficacy profile of GEM in OS-MET-R-092 cells was assessed after 96 h and 7 days of treatment. Sensitivity to single drugs was expressed in terms of IC50 (drug concentration resulting in 50% inhibition of cell growth). IC50 values were calculated from non-linear transformation of dose–response curves using GraphPad Prism 8 (version 8.4.3 Software; RRID:SCR002798).

To assess the in vitro interaction of GEM and DOC, OS cell lines were treated with two distinct schedules: GEM followed by DOC (GEM>DOC) and GEM followed by GEM *plus* DOC (GEM>GEM*plus*DOC). Treatments were applied at constant GEM:DOC ratios optimized for each cell line. Specifically, OS-MET-R-092 cells were treated at a 10:1 ratio, while U-2 OS and SaOS-2 cells were treated at a 3:1 ratio. The type of interaction (synergism, additivity, or antagonism) was determined by calculating the combination index (CI) according to the Chou–Talalay equation [60] using CompuSyn software (RRID:SCR_022931). The ranges of CI reported in CompuSyn software indicated drug–drug interaction as synergistic with CI ≤ 0.90, antagonistic with CI ≥ 1.10, and additive when 0.90 < CI < 1.10.

### 4.10. Flow Cytometry

The expression of CD44 was assessed by flow cytometry. Five hundred thousand (5 × 10^5^) cells were incubated with the monoclonal conjugated anti-CD44-PE diluted 1:200 (monoclonal antibody, rat, anti-human CD44, [IM7], AB218750, Abcam, Waltham, MS, USA; RRID:AB_10680515) for 30 min on ice. Fluorescence was detected using a BD Scientific FACSCanto Flow Cytometer (Becton Dickinson, NJ, USA; RRID:SCR_018055) and data were analyzed with the BD FACSDiva Software (RRID:SCR_001456).

### 4.11. Establishment of OvoPDXs

Fertilized, specific pathogen-free (SPF) chicken eggs were purchased from the Chick Farm Company (Faenza, Italy) and incubated at 37 °C with 56% relative humidity under scheduled rotation. On EDD 5, 2–3 mL of albumen were removed and a window was opened on the eggshell. On EDD 8, OS-MET-092 PDTFs (0.3 × 0.3 cm) were implanted onto the CAM. On EDD 15, embryos were euthanized, and the fragments were excised. Prior to implant and after explant, the fragments were weighed and viability was checked by PrestoBlue (1:10; Invitrogen, Bleiwijk, The Netherlands; A13262). In parallel, control samples (*n* = 12) were maintained in culture medium in 24-well plates without further handling (static conditions).

Explanted PDTFs were fixed in 10% neutral buffered formalin (11699408, VWR) and subsequently embedded in paraffin. Histological sections were stained with H/E using an automated slide stainer.

### 4.12. Statistics

GraphPad Prism (version 7.0 Software; RRID:SCR002798) was employed to perform statistical analysis. Means were compared using Student *t* test or One-Way ANOVA when the experimental data included more than two groups. Dunnett’s multiple comparison test followed the One-Way ANOVA to observe significant differences from the data of OS-MET-R-092 and the other two cell lines, which were identified with the following symbols according to the different *p*-values: (*p* < 0.0001, ****), (*p* < 0.001, ***), and (*p* < 0.05, *).

## Figures and Tables

**Table 1:** STR profiling of the patient tumor sample and OS-MET-R-092 at passage 6 (p6). § indicates partially discordant loci.

Locus	Patient Tumor Sample	OS-MET-R-092 p6
D8S1179	13, 14	13, 14
D21S11	29, 30, 31.2	29, 30, 31.2
D7S820	11, 11	11, 11
CSF1PO	11, 12	11, 12
D3S1358	17, 18	17, 18
TH01 ^§^	6, 7	7, 7
D13S317	9, 9	9, 9
D16S539	11, 11	11, 11
D2S1338	20, 21	20, 21
D19S433	14, 14.2	14, 14.2
vWa ^§^	17, 18, 19	17, 18
TPOX	8, 10	8, 10
D18S51 ^§^	15, 16, 17	16, 17
AMEL	X, Y	X, Y
D5S818	12, 13	12, 13
FGA	20, 26	20, 26

**Table 2:** List of primers and assays employed for gene expression analysis.

Target Name	Forward Primer Seq. 5′-3′	Reverse Primer Seq. 5′-3′	Assay Number
β-actin	ATCGTCCACCGCAAATGCTTCTA	AGCCATGCCAATCTCATCTTGTT	
CD44	CTGCCGCTTTGCAGGTGTA	CATTGTGGGCAAGGTGCTATT	
COL1A1	GCTATGATGAGAAATCAACCG	CATTGTGGGCAAGGTGCTATT	
BMP-2			NM_001200.2
ALP	GGGCTCCAGAAGCTCAACAC	GTGGAGCTGACCCTTGAGCAT	
RUNX-2	TGATGACACTGCCACCTCTGA	GCACCTGCCTGGCTCTTCT	
MMP1	CAAATGGGCTTGAAGCTGCTTACG	GTGTAGCACATTCTGTCCCTGAACA	
MMP2	GCTCCACCACCTACAACTTTGAGAA	TGTCATAGGATGTGCCCTGGAA	
MMP3	AGTCTTCCAATCCTACTGTTGCT	TCCCCGTCACCTCCAATCC	
MMP9	CTTTGAGTCCGGTGGACGAT	TCGCCAGTACTTCCCATCCT	
MMP13	GACTTCCCAGGAATTGGTGA	TGACGCGAACAATACGGTTA	
MMP14	GGCTACAGCAATATGGCTACC	GATGGCCGCTGAGAGTGAC	
ABCC1			Hs00219905_m1
ABCC2			Hs00166123_m1
ACTB			Hs99999903_m1
ATM			Hs00175892_m1
ATR			Hs00992123_m1
ERCC1			Hs01012158_m1
ERCC2			Hs00361161_m1
ERCC3			Hs01554457_m1
ERCC4			Hs00193342_m1
ERCC5			Hs01557031_m1
XPA			Hs00166045_m1

## Data Availability

The original contributions presented in this study are included in the article/Supplementary Materials. Further inquiries can be directed to the corresponding author(s).

## Supplementary Materials

The following supporting information can be downloaded at https://www.mdpi.com/article/10.3390/ijms262110540/s1.

## Abbreviations

The following abbreviations are used in this manuscript:

α-MEM	Alpha-Modified Eagle’s Minimum Essential Medium
ABCB1	ATP Binding Cassette Subfamily B Member 1
ABCC	ATP Binding Cassette Subfamily C Member
ALPL	Alkaline phosphatase
ATM	ATM Serine/Threonine kinase
ATR	ATR Checkpoint kinase
BMP	bone morphogenetic protein
CAM	chorioallantoic membrane
CD31	Cluster of differentiation 31
CD44	Cluster of differentiation 44
CDDP	cisplatin
CECT	contrast-enhanced computed tomography
CI	combination index
COL1A1	Collagen type I α1
CPD	cumulative population doubling
DNA	Deoxyribonucleic Acid
DMEM HG	Dulbecco’s Modified Eagle Medium high glucose
DOC	docetaxel
DT	doubling time
DOXO	doxorubicin
ECM	extracellular matrix
EDD	egg development day
ERCC	ERCC Excision Repair
FBS HI	heat-inactivated fetal bovine serum
GEM	gemcitabine
HGF	Hepatocyte growth factor
H/E	hematoxylin/eosin
IFO	ifosfamide
MAP	methotrexate adriamicin cisplatin
MMP	matrix metalloproteinase
MTX	methotrexate
NER	Nucleotide excixion repair
OS	osteosarcoma
ovoPDX	Patient-derived xenograft in ovo
PBS	phosphate-buffered saline
PD	population doubling
PDCC	patient-derived cell culture
PDTF	patient-derived tissue fragment
qRT-PCR	Quantitative real time polymerase chain reaction
RNA	ribonucleic acid
RUNX2	RUNX Family Transcription Factor 2
SPF	specific pathogen-free
STR	short tandem repeat
ULA	ultra-low attachment
XPA	XPA, DNA Damage Recognition And Repair Factor
