# Supplementary material for: Establishment and Characterization of OS-MET-R-092: A Novel Patient-Derived Cell Culture from an Osteosarcoma Bone Metastasis
Source: Int J Mol Sci. 2025 Oct 29;26(21):10540. doi: 10.3390/ijms262110540 (PMC12609462; doi:10.3390/ijms262110540)
Supplement: Supplementary file 1 [file ijms-26-10540-s001.zip › ijms-3897616-supplementary.pdf]

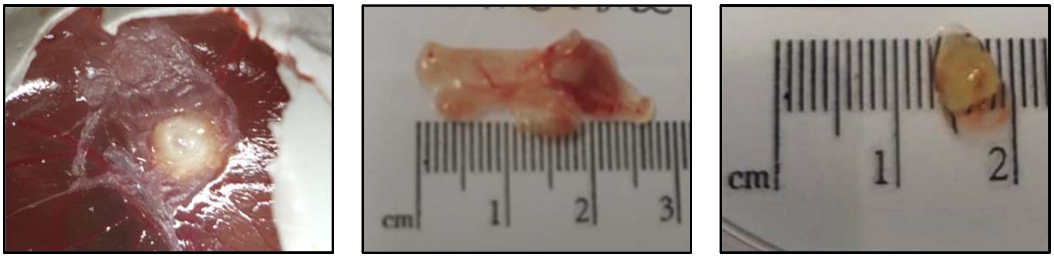

Supplementary Figure S1 OS-MET-092 *ovo*PDX

Photographs of an OS-MET-092 fragment implanted on the CAM membrane (left) and of two representative explants (middle and right).

OS-MET-R-092

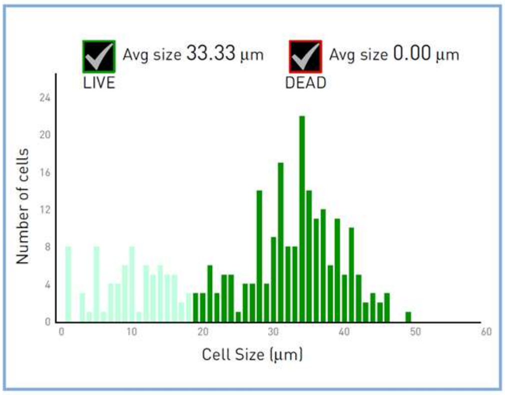

SaOS-2

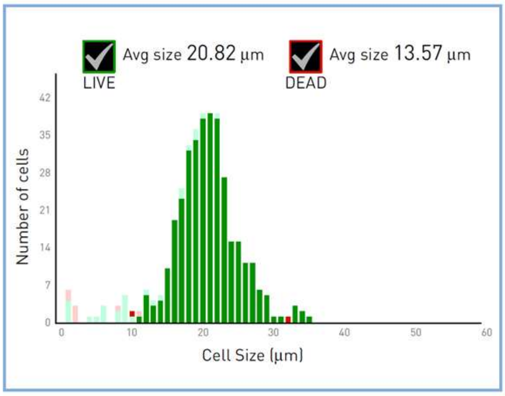

U-2 OS

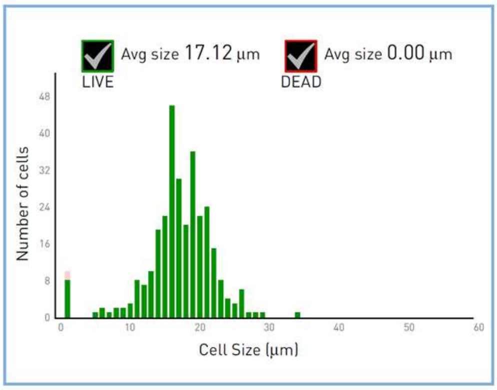

Supplementary Figure S2 Cell size distribution

Average cell size and cell size distribution of OS-MET-R-092, SaOS-2 and U-2 OS measured by Countess II FL Automated Cell Counter.

A

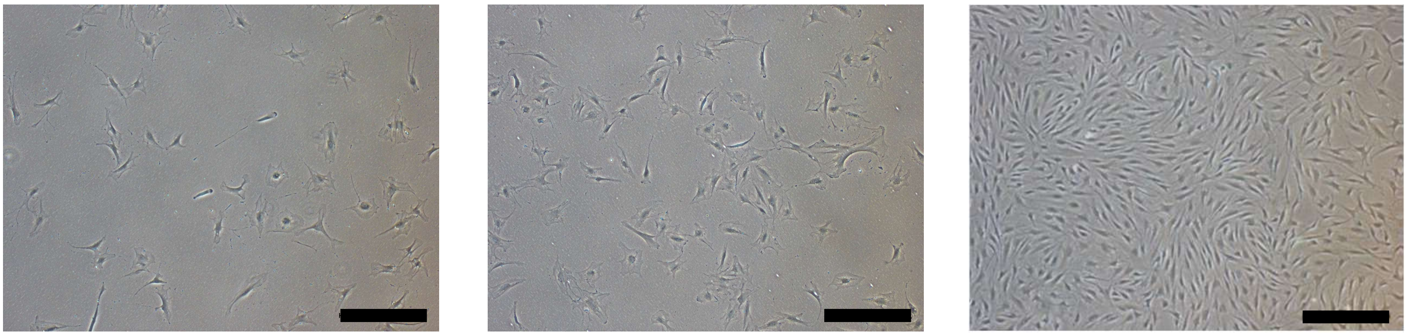

500 cells **1,250 cells** **5,000 cells**

B

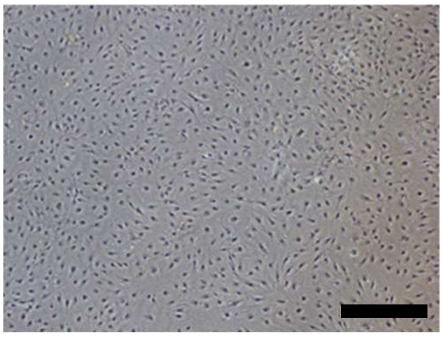

C

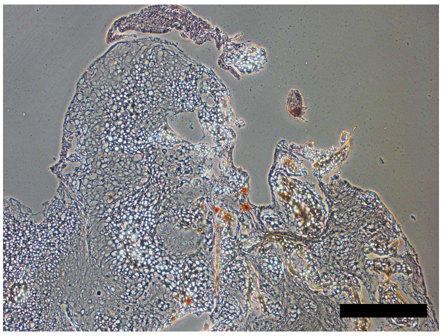

Supplementary Figure S3 Characterization of OS-MET-R-092 cells

A: Micrographs, complete with scalebars (500µm), of OS-MET-R-092 cells seeded in increasing quantities for the evaluation of clonal efficiency.
B: Micrograph of OS-MET-R-092 cells cultured in osteogenic medium before Alizarin Red staining.
C: Micrograph of section obtained from patient specimen stained with Alizarin Red.

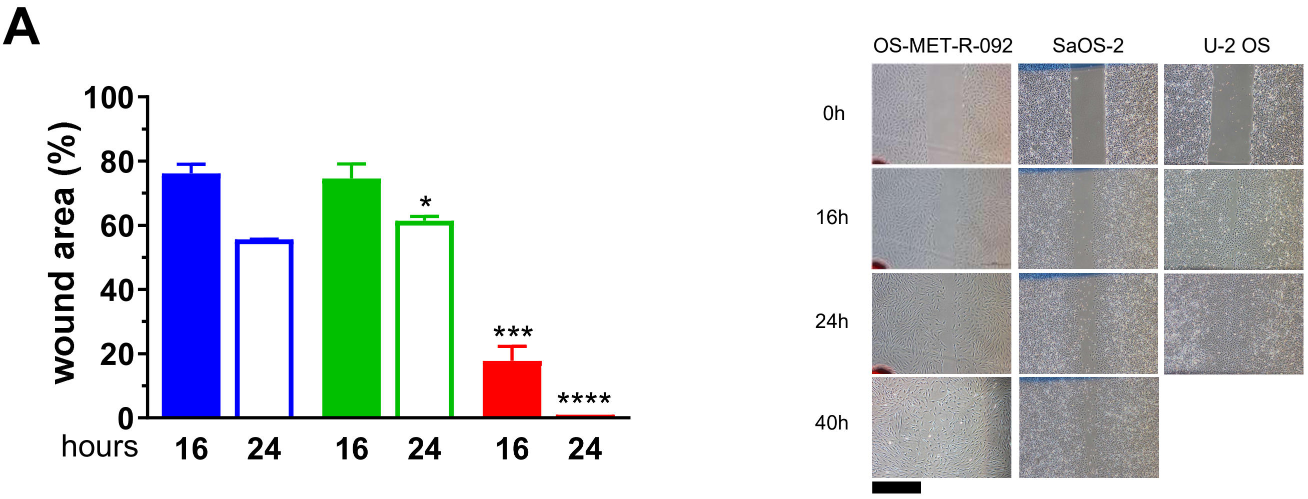

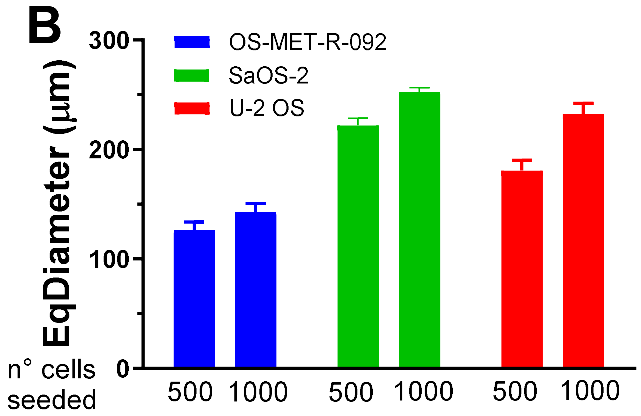

	SHAPE FACTOR		ROUGHNESS	
	500 cells	1000 cells	500 cells	1000 cells
	Mean+SEM	Mean+SEM	Mean+SEM	Mean+SEM
**OS-MET-R-092**	0,868±0,038	0,872±0,039	1,135±0,017	1,134±0,038
**SaOS-2**	0,662±0,049	0,661±0,041	1,460±0,216	1,481±0.237
**U-2 OS**	0,746±0,001	0,739±0,010	1,336±0,019	1,381±0,074

Supplementary Figure S4 Invasive phenotype of OS-MET-R-092 cells

A: Wound healing assay: Left: each bar represents the percentage of residual wound area after 16h (solid) or 24h (empty). Right: Micrographs of representative fields over time. Scale bar = 500µm.

B: Spheroids diameters of OS-MET-R-092, SaOS-2 and U-2 OS at day 7. The table reports the relative values of shape factor (round = 1) and roughness (smooth = 1).

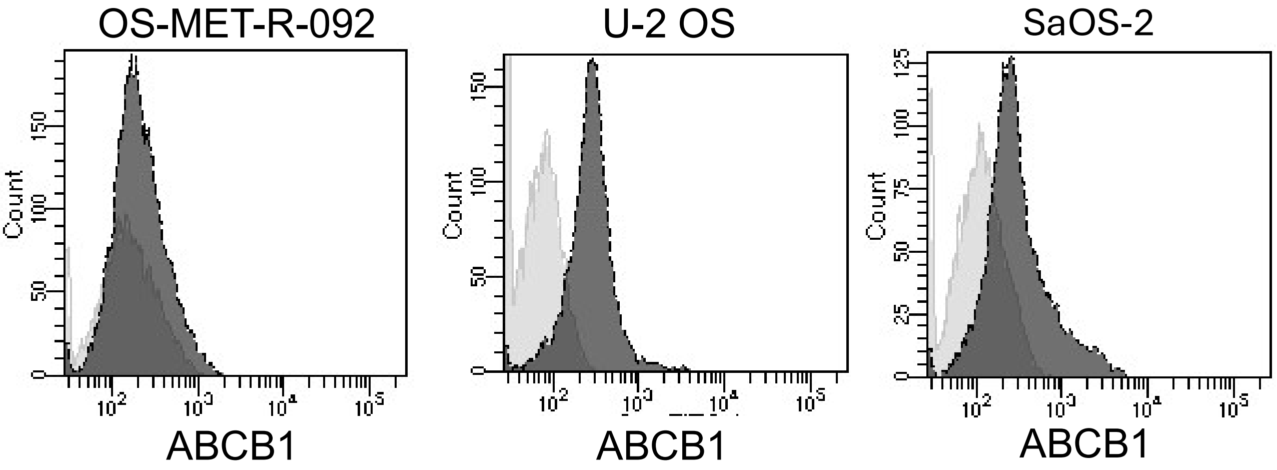

Supplementary Figure S5 Expression of ABCB1

Cytofluorimetric analysis of ABCB1 expression. The overlays show the profile of unstained cells in light grey and stained ones in dark grey.

Supplementary methods

1. Alizarin Red Staining

Paraffin-embedded tissue sections were stained with Alizarin Red to detect calcium deposition. After passages in xylene and alcohols, sections were incubated with Alizarin Red for two minutes, dehydrated with acetone 100% and then with acetone:xylene 1:1. Sections were then mounted and observed.

2. Wound healing assay

Cells (1×10^5) cells were seeded in 6-well plates and incubated until confluence. The monolayer was scratched with a P200 pipette tip. The wound closure was monitored by capturing images at 0, 16, 24, and 40 hours post-scratch at 40× magnification. The wound area was quantified using the area auto-detection tool of the NIS-Elements Basic Research software. The experiment was performed in technical triplicate and biological duplicate.

3. Flow cytometry

The expression of ABCB1 was assessed by flow cytometry. Cells (5×10^5) were fixed in 4% paraformaldehyde (Sigma-Aldrich, P6148) and permeabilized with 0.1% saponin (Sigma-Aldrich, 47036). Cells were incubated with the primary anti-ABCB1 antibody (Anti-P-glycoprotein antibody [clone number MRK16], MBS488127, MyBioSource Inc., mouse)and subsequently with the Alexa Fluor® 488-conjugated secondary antibody (Life Technologies, A11059, rabbit, RRID:AB_2534106), both diluted 1:100 in 0.1% saponin. Fluorescence was detected using a BD Scientific FACSCanto Flow Cytometer (Becton Dickinson, RRID:SCR_018055) and data were analyzed with the BD FACSDiva Software (RRID:SCR_001456).
